# A Successful Live Birth After Double Fertility Preservation With Embryo Cryopreservation and MPA Therapy Combined With Hysteroscopic Resection for Metachronous Breast and Endometrial Cancer in Women With Cowden Syndrome: A Case Report

**DOI:** 10.1002/rmb2.70035

**Published:** 2026-03-05

**Authors:** Itsuki Kajimura, Michio Kitajima, Shoko Miura, Chiaki Eishi, Riko Matsuda, Kanako Matsumoto, Ayumi Harada, Yuriko Kitajima, Yuri Hasegawa, Megumi Matsumoto, Keitaro Matsumoto, Kiyonori Miura

**Affiliations:** ^1^ Department of Obstetrics and Gynecology Nagasaki University Hospital Nagasaki Japan; ^2^ Woman's Medical Center, Takagi Hospital, Department of Obstetrics and Gynecology International University of Health and Welfare Okawa Japan; ^3^ Clinical Genomics Center Department of Obstetrics and Gynecology Nagasaki University Hospital Nagasaki University Hospital Nagasaki Japan; ^4^ Division of Surgical Oncology, Department of Surgery Nagasaki University Graduate School of Biomedical Sciences Nagasaki Japan

**Keywords:** breast cancer, Cowden syndrome, endometrial cancer, fertility preservation, hereditary tumors

## Abstract

**Case:**

Cowden syndrome is an autosomal‐dominantly inherited rare condition caused by germline pathogenic variants of the *PTEN* gene. Multiple tumor development at a younger age in this syndrome may warrant different modalities of fertility preservation. Here we report that women with this syndrome gave successful live birth with frozen–thaw embryo transfer that was cryopreserved before the start of chemotherapy for breast cancer. This woman also suffered from endometrial cancer that developed during hormonal treatment with GnRH agonist and tamoxifen for breast cancer. The lesion was well controlled with oral MPA therapy and hysteroscopic resection.

**Outcome:**

She could receive fertility preservation for breast cancer and endometrial cancer before fertility treatment and pregnancy.

**Conclusion:**

This is the first report of live births after multiple fertility preservation therapy for metachronous breast and uterine cancer in Cowden syndrome. Appropriate genetic counseling may help the patient's decision making. The treatment plan should be discussed with diverse professionals such as oncologists, reproductive physicians, perinatologists, geneticists, and genetic counselors.

## 1 Introduction

Cowden syndrome is a rare autosomal‐dominantly inherited condition caused by germline pathogenic variants in *PTEN* [1]. Women with Cowden syndrome are more likely to develop cancers such as breast, thyroid, endometrial, colorectal, and renal cancers at a relatively younger age [2]. Fertility preservation is recommended before gonadotoxic cancer treatment [3]. Especially for women with hereditary tumors, we should pay attention to multiple cancer development and provide them an appropriate genetic counseling. Here we report a woman with Cowden syndrome who received fertility preservation for breast cancer and MPA therapy for endometrial cancer that developed during adjuvant treatment for breast cancer and achieved a successful live birth with frozen–thaw embryo transfer that was cryopreserved before cancer treatment.

## 2 Case Report

Thirty‐two‐year‐old nulliparous woman, married at age 31 without family history of breast cancer or other medical conditions was referred to our outpatient clinic.

She had been aware of breast masses since she was 16 years old, and was followed up by the breast surgery department with a diagnosis of bilateral juvenile multiple papillomatosis. At age 22, a benign lipoma and hemangioma of the knee joint were removed, and at age 31, a vascular embolization procedure was performed for a right subclavian arteriovenous malformation. In addition, an adenomatous goiter was observed in the thyroid gland, and macrocephaly was noted.

**FIGURE 1:**
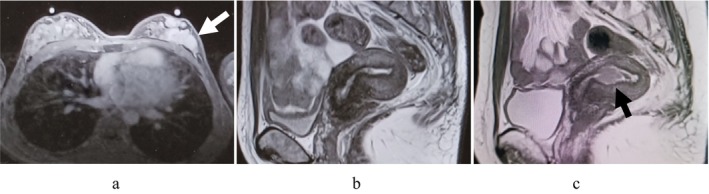
(a) Breast MRI image (T1‐weighted transverse view). 38 × 31 × 18 mm mass with irregular margins is seen in the C region of the left breast (white arrow). (b) Pelvic MRI scan showed a lesion in the uterine lumen with mixed mild low and high signal on T2‐weighted images. (c) Pelvic MRI scan shows an 18‐mm mass lesion in the uterine lumen with mild high signal on a well demarcated T2‐weighted image (black arrow).

Above mentioned clinical findings suggested the possibility of a genetic disease such as Cowden syndrome, and she was referred to our breast and endocrine surgery department at the age of 31 for detailed inspections. Genetic counseling was conducted and her genetic testing revealed a pathological variant of the *PTEN* gene (NM_000314.4:c.37A>T(p.Lys13*)[heterozygous]), leading to a diagnosis of Cowden syndrome. No cases of breast cancer or multiple tumors suggestive of Cowden syndrome were identified within her family history up to the third degree of kinship, including her parents and younger sister. A de novo mutation was suspected. On the way of checkup, she was diagnosed with breast cancer by biopsy from a left mammary mass and underwent left total mastectomy and axillary lymph node dissection at the age of 32 (Figure 1a). Finally, she was diagnosed as stage IIIA left breast cancer (pT3N2aM0, ER positive, PgR positive, HER2 negative). Postoperative chemotherapy (EC therapy: epirubicin and cyclophosphamide, DTX therapy: docetaxel alone), radiation therapy, and hormone therapy (LH‐RH agonist and tamoxifen) were planned.

Before postoperative adjuvant treatment, the patient was referred to our department for consultation regarding fertility preservation. The risk of gonadotoxicity of chemotherapy for breast cancer was predicted to be intermediate risk based on ASCO guidelines [4], and the risk of decreased fertility due to long‐term hormone therapy was also considered. Considering the risk of offsprings, genetic counseling was provided again. The clinical manifestations of the disease, the risk of tumor development, and the need for continuous surveillance, as well as the possibility of inheritance to the next generation (1/2 chance of inheriting the same pathological variant) due to the autosomal manifestations of the disease had been intensely discussed. If a pathological variant is inherited by the child, surveillance is recommended starting in adolescence, as in this case, and multiple treatments may be required if a tumor develops. To avoid such risks, preimplantation genetic testing for monogenic disease (PGT‐M) is an option. Through reproductive and genetic counseling, she requested fertility preservation and chose embryo freezing, but did not request PGT‐M. At her first visit (menstruation day 14), hormone profiles showed FSH 8.52 mIU/mL, LH 8.36 mIU/mL, E2 90.8 pg/mL, P4 0.74 mIU/mL, and AMH 4.37 ng/mL. The random‐start ovarian stimulation was selected, and self‐administration of follitropin alfa and GnRH antagonist was started on menstruation day 17. An oral aromatase inhibitor (letrozole) was co‐administrated. A total of 14 growing follicles were found in both ovaries. Follicle aspiration was performed 12 days after the start of ovarian stimulation. Thirteen eggs were retrieved, and 9 of them were fertilized by conventional IVF. On days 5 and 6 after egg retrieval, five blastocysts with good morphology, evaluated by Gardner's classification, were cryopreserved by the vitrification method at the age of 32.

**FIGURE 2:**
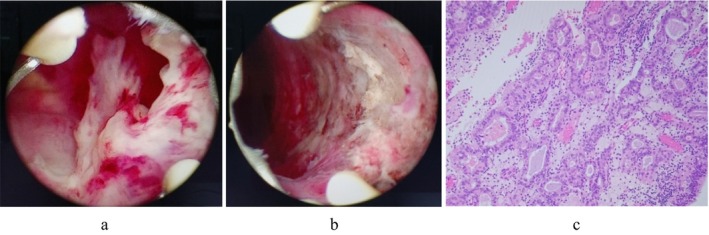
Hysteroscopic surgery findings (a,b) and pathological findings (c). (a) A polypoidal mass growing from the left lateral wall of the uterine lumen after MPA therapy. (b) Lumen of the uterus after resection with bipolar resectoscope. (c) Proliferation of atypical endometrial glandular epithelium with glandular duct structures (hematoxylin and eosin staining, 100×).

Chemotherapy for breast cancer was started 20 days after egg retrieval, with 4 courses of dose‐dense EC therapy, followed by 4 courses of DTX therapy. Two months after egg retrieval, the patient had persistent abnormal uterine bleeding during chemotherapy. Transvaginal ultrasonography revealed a 1‐cm polyp‐like lesion in the uterus, but endometrial cytology was negative for malignancy. Pelvic MRI scan showed a lesion in the uterine lumen with mixed mild low and high signal on T2‐weighted images, which was either negative for malignancy (Figure 1b). Thereafter, abnormal uterine bleeding disappeared and there was no increase in endometrial lesions. After the completion of chemotherapy, LH‐RH agonist and oral tamoxifen therapy was started. After 10 months, the patient again became aware of abnormal uterine bleeding, and a transvaginal ultrasound showed a stable polyp lesion in the uterine cavity and endometrial cytology was again negative for malignancy. We repeatedly performed transvaginal ultrasonography six months later, which revealed the solid mass had enlarged to 2 cm in size. The endometrial aspiration biopsy was diagnosed as atypical endometrial hyperplasia/endometrial intraepithelial neoplasia. An additional pelvic contrast‐enhanced MRI scan was performed, which revealed an 18 mm mass lesion in the lumen of the uterine body, which was suspected to be uterine cancer (Figure 1c). No myometrial invasion was found, and CT scan showed no distant metastases. After the discussion with their breast surgeon, tamoxifen citrate was discontinued and switched to combination therapy of LH‐RH agonist and letrozole. To avoid frequent mechanical interventions to the uterine cavity, we selected primary high‐dose MPA therapy (medroxyprogesterone acetate 600 mg/day) combined with histological endometrial inspections with office hysteroscopy as initial therapy. However, since the lesions persisted even after three months of treatment, complete resolution with MPA therapy alone was likely to be difficult or time‐consuming. As the patient had discontinued breast cancer treatment, hysteroscopic resection under general anesthesia was selected to enable sufficient excision of the lesion. After 15 weeks of MPA therapy, a hysteroscopic endometrial mass resection was performed. A polypoidal mass growing from the left lateral wall of the uterine lumen was removed using a bipolar resectoscope (Figure 2a,b). The pathology of the resected mass was endometrial carcinoma G1 (Figure 2c), and MPA therapy was continued after surgery. Endometrial curettage was performed after an additional 5 weeks of treatment, and no malignant findings were observed.

GnRH agonist and letrozole were resumed, but four months later the couple wished to begin fertility treatment. After discussing with the attending breast surgeon, the hormonal therapy was discontinued. The serum AMH value was as low as 0.03 ng/mL, but regular menstruation resumed 3 months after the end of hormone therapy. At the age of 36, frozen–thawed embryo transfers in natural ovulatory cycles using cryopreserved blastocysts before breast cancer treatment were preferred. Pregnancy was achieved on the fifth embryo transfer. Without perinatal complications, a 2,928 g female baby was delivered vaginally at 40 weeks of gestation. Three months after delivery, she resumed taking letrozole. As for endometrial cancer, transvaginal ultrasonography showed no abnormal findings in the uterine lumen, and endometrial cytology was negative. The patient is free from recurrence with continuous hormonal therapy and close observation.

## 3 Discussion

Cowden syndrome is a rare autosomal‐dominantly inherited condition caused by germline pathogenic variants in *PTEN*. It is associated with multiple hamartomatous lesions occurring in various organs and tissues, including the gastrointestinal tract, skin, mucous membranes, breast, thyroid, endometrium, and brain [1]. Cowden syndrome is diagnosed according to the National Comprehensive Cancer Network (NCCN) clinical diagnostic criteria [5, 6]. If the clinical diagnosis is met, risk assessment and genetic counseling should be provided. The prevalence of the pathological variants of *PTEN* in patients who meet the clinical diagnostic criteria for this syndrome is reported to be 30% to 92% [1].

Cowden syndrome is prone to malignancies such as breast, thyroid, endometrial, colorectal, and renal cancers [2, 6]. Breast cancer is the most frequent type of malignancy in this syndrome. In this report, the patient developed breast cancer at the age of 32 and uterine cancer at the age of 33, and both occurred at a young age. Particularly elevated prevalence of breast cancer in females with *PTEN* mutations is noted, beginning around the age of 30 and rising to an estimated 85% lifetime risk [2]. On the other hand, *PTEN‐*related endometrial cancer risk begins at the age of 25 rising to 30% by the age of 60 [2]. Cases of uterine cancer in Cowden syndrome have been reported in individuals as young as 14–15 years [7, 8]. In addition, elevated risks of thyroid (51.1%), colorectal (10.3%), and kidney cancers (30.6%) were found [2].

Appropriate surveillance for early diagnosis of associated cancers is required because patients have a high risk of multiple cancers in Cowden syndrome [9]. In the NCCN guidelines, breast awareness starting at the age of 18 years, and clinical breast examination every 6–12 months starting at the age of 25 years, or 5 to 10 years before the earliest breast cancer diagnosis in the family (whichever comes first) is recommended [4]. Annual mammogram with consideration of tomosynthesis (3D mammography) and breast MRI with contrast at the age of 30–35 years, or 5–10 years before the earliest breast cancer diagnosis in the family (whichever comes first) is also recommended [4]. For endometrial cancer screening, patient education and prompt medical attention to symptoms (abnormal or postmenopausal uterine bleeding), and consider starting endometrial cancer screening by the age of 35 years [4]. If surveillance for endometrial cancer is offered it should be as part of a clinical trial, but endometrial biopsy every 1–2 years can be considered [4, 8]. In the surveillance of *PTEN* Hamartoma Tumor Syndrome, incorporating endometrial biopsy with annual transvaginal ultrasound may enhance the detection of premalignancies [10]. Cowden syndrome is a rare disease, and this surveillance guideline should be communicated to all oncologists.

It is important to consider fertility preservation for young cancer patients. Health care providers should initiate the discussion on the possibility of infertility with patients with cancer treated during their reproductive years as early as possible [3]. In females, oocyte, embryo, and ovarian tissue cryopreservation are considered according to individual preferences, and conservative therapy is considered for cancers of the female reproductive tracts. In Cowden syndrome, there is a risk of developing malignancy at an age when fertility is a major consideration, and there may be frequent opportunities to provide discussion on fertility preservation. However, there are no established guidelines and specific recommendations for fertility preservation for hereditary tumors. Nulliparous women with Cowden syndrome complicated by breast cancer and endometrial polyps were reported but fertility preservation had not been performed in this case [11]. It is important to detect cancer earlier through meticulous surveillance and be aware of the development of other cancers even after the fertility preservation. In this present case, the patient developed metachronous breast cancer and uterine cancer at a young age just after the marriage, and fertility preservation therapy for each disease had to be considered. Embryo cryopreservation was successfully accomplished before the start of chemotherapy and frozen–thaw embryo transfer was preferred on the way of long‐term hormonal therapy which end up with successful live birth. These procedures might not have interfered with cancer prognosis; however, long‐term follow‐up may be required.

Fertility‐sparing therapies for early‐stage endometrial cancer include oral progestins (megestrol (MA) or medroxyprogesterone acetate (MPA)), levonorgestrel‐releasing intrauterine device (LNG‐IUS), anti‐estrogen treatment, and hysteroscopic resection of the endometrium. These treatments may be combined [10, 12]. MPA and hysteroscopic resection of tumors were effective in the present case. According to ESGO/ESHRE/ESGE Guidelines for the fertility‐sparing treatment of patients with endometrial carcinoma, a combined approach consisting of hysteroscopic tumor resection, followed by oral progestins and/or levonorgestrel intra‐uterine device, is the most effective fertility‐sparing treatment [13]. In this case, performing hysteroscopic surgery first was also an option, but to avoid frequent mechanical interventions to the uterine cavity, we selected primary high‐dose MPA therapy. The prognosis of sporadic endometrial cancers with somatic *PTEN* mutation is relatively good, although currently no different treatment strategy for endometrial cancers is adopted between this syndrome and sporadic patients [1]. In Cowden syndrome, there have been cases in which a total hysterectomy was performed even in cases of young onset [7, 8], and a case underwent dilation and curettage (D&C) for atypical endometrial hyperplasia that developed during breast cancer treatment [14, 15]. However, there are no reports of MPA therapy combined with hysteroscopic surgery for endometrial cancer developed in women with this syndrome.

In addition to fertility preservation therapy, genetic counseling regarding inheritance to the next generation is also important in hereditary tumor syndrome. In autosomal dominant inheritance, affected individuals have a 50% chance of transmitting their pathogenic variant to their children. As for the treatment plan for Cowden syndrome, including fertility preservation, therapy should be discussed in collaboration with genetic specialists and genetic counselors. Additionally, PGT‐M is considered for hereditary cancer syndromes [16]. In this case, the risk of developing tumors from a young age and the need for multiple surgeries and other treatments place a significant burden on patients, and the same risk is passed on to the child. Accordingly, PGT‐M is a valuable option; however, it is also considered that surveillance and treatment may be sufficient, and PGT‐M may raise ethical concerns. In Japan, PGT‐M is offered for severe hereditary diseases under strict candidate selection, and while no cases have been performed for this disorder at this time, genetic counseling requires that it be presented as an option. If necessary, multiple counseling should be conducted over the course of treatment. In the present case, we provided multiple genetic counseling sessions with different specialized personnel, which might aid decision making on fertility preservation and subsequent fertility treatment.

Considering the postpartum treatment plan for this case, close monitoring for tumor development is provided regularly. In addition to resuming hormone therapy for breast cancer, she requires follow‐up for endometrial cancer recurrence. In the ESGO/ESHRE/ESGE Guidelines, clinical pelvic examination and ultrasound scan at every 3‐month follow‐up visit, and endometrial histological assessment should be performed every 3–6 months by hysteroscopy according to the results of imaging [13]. Furthermore, regular thyroid ultrasounds, colonoscopies, and kidney CT scans are recommended for monitoring the development of other tumors associated with Cowden syndrome [1]. About the additional infertility treatment in this case, if she wishes for a second child, another egg retrieval must be performed despite diminished ovarian reserve. Infertility treatment may take time, carrying the risk of tumor recurrence or new tumor development. As she did not wish to continue further infertility treatment, definitive surgery for endometrial cancer at an early stage was performed. Therefore, it is necessary to continue having thorough discussions with her and her family.

We performed a comprehensive literature search on fertility preservation and Cowden syndrome; however, no English literature has been found reporting the cases with successful fertility preservation after the development of breast or endometrial cancer in women with PTEN gene mutation. Neither reproductive nor obstetrical outcomes in this syndrome have been well examined. Cowden syndrome is a hereditary tumor disorder and carries a risk of developing multiple tumors during reproductive years, making it crucial to develop cancer treatment strategies that include fertility preservation and genetic counseling.

## 4 Conclusion

This is the first report of live births after multiple fertility preservation therapy for metachronous breast and uterine cancer in Cowden syndrome. As in this case, multiple cancer treatments, different modalities of fertility preservation, and meticulous genetic counseling were important for successful pregnancy. The treatment plan should be discussed with diverse professionals such as oncologists, reproductive physicians, perinatologists, geneticists, and genetic counselors.

## Funding

No funding was received for conducting this study.

## Disclosure

The participant has consented to the submission of the case report to the journal. The Ethics Committee of Nagasaki University Hospital has confirmed that no ethical approval is required.

## Conflicts of Interest

The authors declare no conflicts of interest.

## Data Availability

The data that support the findings of this study are available from the corresponding author upon reasonable request.
